# Parenting behaviour is highly heritable in male stickleback

**DOI:** 10.1098/rsos.171029

**Published:** 2018-01-10

**Authors:** Alison M. Bell, Rebecca Trapp, Jason Keagy

**Affiliations:** 1 School of Integrative Biology, University of Illinois at Urbana—Champaign, Urbana, IL 61801, USA; 2 Carl R. Woese Institute for Genomic Biology, University of Illinois at Urbana—Champaign, Urbana, IL 61801, USA; 3 Program in Ecology, Evolution and Conservation, University of Illinois at Urbana—Champaign, Urbana, IL 61801, USA; 4 Program in Neuroscience, University of Illinois at Urbana—Champaign, Urbana, IL 61801, USA; 5 Department of Animal Biology, University of Illinois at Urbana—Champaign, Urbana, IL 61801, USA

**Keywords:** paternal care, parental care, three-spined stickleback, *Gasterosteus aculeatus*, animal model, genetic variation

## Abstract

Parental care is critical for fitness, yet little is known about its genetic basis. Here, we estimate the heritability of parenting behaviour in a species famous for its diversity and its behavioural repertoire: three-spined stickleback (*Gasterosteus aculeatus*). Male three-spined stickleback are the sole providers of parental care that is necessary for offspring survival; therefore, this system offers the opportunity to study the inheritance of parental behaviour when selection is primarily acting on males. Fanning behaviour is a conspicuous parental behaviour that is readily quantified in this species. We show that the heritability of fanning behaviour is ≥0.9 and significantly different from zero within a freshwater population. Moreover, there was abundant genetic variation for fanning behaviour, indicating that it could readily evolve. These results suggest that parenting behaviour is tractable for further genetic dissection in this system.

## 1. Introduction

For many organisms, providing parental care is necessary for the survival of offspring [1]. Different forms of parental care have evolved and organisms exhibit a wide array of behaviours associated with offspring defence, brooding and provisioning. Given the importance of parenting behaviour for fitness and its diversity, it is surprising that we know so little about its genetic basis.

A few studies in birds and insects have estimated the heritability of parenting behaviours associated with offspring provisioning [2–7]. Other studies have focused on the heritable basis of indirect forms of parental care such as oviposition behaviour in insects and reptiles [8–11], and nest construction in mammals [12,13]. A recent study identified regions of the genome harbouring genetic variation for interspecific variation in paternal behaviour in monogamous *Peromyscus* mice [14]. However, we know little about the inheritance of paternal behaviour in systems where fathers are the only caregivers. When both mothers and fathers provide parental care, selection acting on maternal behaviour might inadvertently influence paternal behaviour, and vice versa. However, when only one sex (e.g. males) provides care, then selection is potentially less constrained {Walling, 2008 #20} [7]. Moreover, despite tremendous diversity in parenting behaviour among fish species [15], the genetic basis of parenting behaviour in fishes is unknown.

Three-spined stickleback fish (*Gasterosteus aculeatus*) are a model species in behaviour and evolution and are suitable subjects for studying the genetic basis of parenting behaviour because they exhibit tremendous repeatable variation in parental care among individuals within populations [16,17]. In this species, males are the sole providers of parental care; therefore, this system offers the opportunity to study the inheritance of parental behaviour when selection is primarily acting on males. During the breeding season, male stickleback defend territories, construct nests and attract females to spawn in the nest. After spawning, males provide all of the parental care for the offspring that is necessary for offspring survival. Males defend the nest from nest predators and engage in high levels of conspicuous parenting behaviours such as fanning and tending the nest. After the eggs hatch, fathers continue to defend their newly hatched and vulnerable fry from predators. They also chase and retrieve their free-swimming fry [16]. Fanning is an especially critical parenting behaviour that serves to oxygenate the eggs and remove carbon dioxide; fanning occupies roughly 40% of males' time during its peak [16]. Parental care is energetically costly [18,19] and males adjust their care in response to their diet [20], their mate [21], algal blooms [22], temperature [23], oxygen availability [20], the number of eggs in the nest [24] and embryonic development [16,17,21,23]. Despite this plasticity, individual males consistently differ from one another in parenting behaviour. Individuals consistently differ in the construction of their nests [25] and individual differences in fanning behaviour are repeatable within and across clutches, both in the field and in the laboratory [16,17].

In this study, we assess whether variation in a key parenting behaviour—fanning behaviour—has a heritable component in stickleback. We focus on fanning because it occurs at a high frequency, is readily characterized with a clear beginning and end, has a clear function (to oxygenate embryos) and is essential for embryonic survival. We estimate the heritability of fanning rate by examining the phenotypic resemblance between fathers and sons in a common garden experiment using an animal model. When relatives strongly covary, narrow sense heritability (*h*^2^, additive genetic variation divided by total phenotypic variation) is high, and additive genetic effects contribute substantially to the variation in the trait [26,27]. We also report an estimate of the coefficient of genetic variation (CV_A_), a measure of evolutionary potential that is standardized by the trait mean rather than trait variance and is therefore often a better measure of evolvability than heritability [28].

## 2. Material and methods

To investigate the heritability of parenting behaviour in three-spined stickleback, we measured the parenting behaviour of wild-caught males and their laboratory-reared sons in a common garden environment. Stickleback were collected as breeding adults (approx. 1 year old) from Putah Creek, CA in summer 2015 and maintained in the laboratory in mixed-sex group tanks (108 L × 33 W × 24 H cm) at 20°C on a 16 : 8 photoperiod. The relatedness among wild-caught animals is unknown. When a male showed signs of nuptial coloration (red throat, blue eyes and territorial behaviour), he was measured for standard length with a ruler (mm) and weighed (mg) before being placed in a separate nesting tank (35.5 L × 21.6 W × 19 H cm), which contained a refuge (black plastic plant), a nesting dish filled with sand and string algae for building a nest. The condition was estimated as weight/length. The sides of the tank were covered with black blinds to prevent males from seeing each other. Males were fed once a day with a slurry of defrosted adult brine shrimp (*Artemia* sp.), mysis shrimp (*Mysis* sp.), bloodworms (*Chironomus* sp.) and Cyclop-Eeze. Females were fed twice a day with the same food as males to encourage them to become gravid.

After a male completed his nest as assessed by the characteristic tunnel [29], a gravid female was weighed and introduced into his tank. If the pair spawned, the female was removed after spawning and weighed again. The difference in weight gave us a measure of clutch size. An additional blind was fastened to the front of the male's tank in order to minimize disruption to the parental male. If the pair did not spawn within 2 h, the female was removed and the male was presented with another female on a different day.

Wild-caught males’ parenting behaviour was observed on two occasions: on the day after spawning (egg day 1, ED1) and 5 days after spawning (ED5), which is the day before the eggs typically hatch in this population. The following parenting behaviours were recorded from behind a blind for 5 min: the number of pokes at the nest, time spent at the nest and time spent fanning the nest.

Fathers were removed on ED6; therefore, sons received parental care while they were embryos, but not as fry. We elected to allow the fathers to care for their offspring for a portion of offspring development so that we could measure the fathers' parenting behaviour. Cross-fostering would have been useful to control for non-genetic effects of fathers on offspring and has been used in one other study in stickleback [30], but male stickleback frequently recognize and cannibalize offspring that are not their own, and we suspect that highly attentive fathers are more likely to reject foreign eggs, which could lead to biased sampling [31,32]. Studies on other organisms have suggested that the way that parents care for their offspring might influence offspring behavioural development, thereby introducing environmental, non-genetic effects of parents on offspring that could increase the resemblance between parents and offspring [33,34], but there is no evidence that parenting behaviour is influenced by embryonic experience.

After the father was removed from the tank, the fry (hereafter referred to as F_1_) were reared in the laboratory until adulthood. Initially, fry were fed hatched *Artemia* nauplii and Cyclop-Eeze once a day. As fry became larger, we gradually transitioned them to the same food we fed adult stickleback.

The F_1_ population was reared under winter conditions for four months (15°C, 8 : 16 photoperiod). Then, the temperature and photoperiod were changed to simulate summer (20°C on a 16 : 8 photoperiod). The parenting behaviour of the F_1_ males was measured as described above with the following modifications in order to try to minimize disruptions caused by the observer and improve the sample size. Reproductively mature F_1_ males were kept in larger individual nesting tanks (36 L × 33 W × 24 H cm) and parenting behaviour was measured via a mirror situated over the tank. The parenting behaviour of the F_1_ males was recorded on ED1, ED3, ED5, 1 day after hatching (fry day 1, FD1), FD3 and FD5. We ultimately ended up with a sample size of *n* = 26 F_1_ males from *n* = 13 original fathers that completed the entire experiment (built nests, mated with females and cared for their offspring).

### 2.1. Statistical analysis

**Figure 1.**
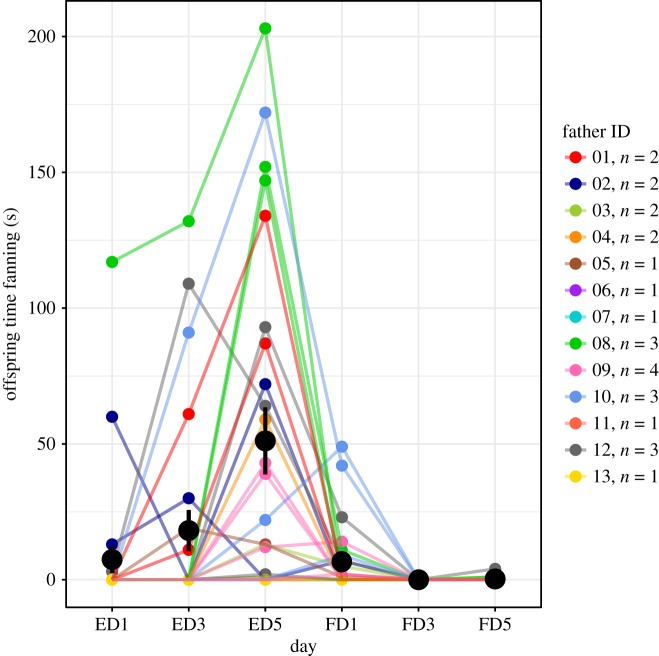
Son fanning behaviour over time. Fanning increased until fry hatched on day 5 and then decreased. Lines indicate traces of individual behaviour over time, and colour coded by family. Large black circles and lines indicate mean ± s.e.

All statistical analyses were done in R v. 3.3.2 [35]. Initial analyses indicated that there was little variation among families in non-fanning nesting behaviour; therefore, we focus on fanning behaviour here. We had data for both fathers and sons for ED1 and ED5. Most (12/13 fathers, 22/26 sons) males did not fan on ED1 (offspring family average = 5.88 s); therefore, we focused on ED5, which corresponds to a peak in fanning (see Results; figure 1) for our analyses.

To test for how fanning changed over time in the sons (for whom we had data covering the entire parenting period), we used a linear mixed-effects regression model using the *lmer* function in the R package *lme4* v. 1.1-12 [36] and tested statistical significance by calculating degrees of freedom using the Satterthwaite approximation with the R package *lmerTest* v. 2.0-33. Because fanning increases over time and then falls back down to zero, we included both a linear effect of day and a quadratic effect of day as fixed effects. We also allowed each fish to have its own intercept and slope for the fixed effects. We used AIC and model comparison tests to confirm that this model was best compared to simpler models.

We used a Bayesian method, Markov chain Monte Carlo simulations, with the R package *MCMCglmm* v. 2.24 [37] to calculate heritability using a variance component approach with a univariate animal model [38]. This method is preferable to parent–offspring regression because it uses information about the relatedness between fathers and sons as well as between brothers, and because it allows us to more easily correct for potential confounding variables. We used weakly informative inverse-gamma priors for the ‘residual’ and ‘genetic’ effects (by setting the MCMCglmm parameters *V* = 1, nu = 0.002). The posterior distribution was sampled every 2000 times (thinning interval), following a burn-in period of 100 000 iterations with a total run of 10 000 000 iterations. These values were chosen to reduce autocorrelation to acceptable levels (less than 0.1). We also verified convergence with a Heidelberg stationarity test. We then divided the genetic variance by the total phenotypic variance to estimate heritability [38] and calculated both a point estimate and a 95% credible interval. Because animals were measured in a common garden in the laboratory, we controlled for many environmental effects such as diet and oxygen availability. However, there were some environmental effects that might influence our heritability estimate; therefore, we also estimated heritability after correcting for the effects of male condition (weight/length, fixed effect), clutch size (fixed effect) and rearing environment (‘patch’, random effect) on fanning behaviour. We used weakly informative inverse-gamma priors for the ‘residual’, ‘genetic’ and ‘patch’ effects (by setting the MCMCglmm parameters *V* = 1, nu = 0.002). We had 10 000 000 iterations with a burn-in of 100 000 iterations and sampled the posterior distribution every 3000 iterations.

To address the possibility that stochastic variance during the short 5 min observation period might have influenced our heritability estimate, in a separate study we sampled five different males for 5 min two times within a 20 min period. We then used MCMCglmm to estimate repeatability of fanning time in a model with just an intercept and male ID as a random factor. The prior was set to split the variance evenly between the intercept and the random factor. We had 500 000 iterations with a burn-in of 5000 iterations, and we sampled the posterior distribution every 100 iterations. We estimated repeatability as the variance explained by individual ID divided by the total variance.

To further explore the possibility that sampling error might have influenced our heritability estimate, we recorded the behaviour of one male for 1 h and sampled at 5 min intervals, giving us a distribution of deviations for a 5 min sample from the proportion of time fanning occurred across the entire hour. We used these data to simulate deviations that may occur due to sampling error and added these deviations to each data point under the condition that fanning was not allowed to be less than 0 or greater than 300 s. We then recalculated the heritability estimate from the animal model. This was then repeated 1000 times to give a range of heritability estimates expected if the deviations were as large as those observed in our 11 five-minute sessions. Because of issues with the computational complexity of running 1000 iterations of an animal model with 10 000 000 iterations, we used the MCMCglmm defaults of 13 000 iterations with a burn-in period of 3000 iterations and sampling of the posterior distribution every 10 iterations.

We estimated two measures of evolvability (*I*_A_ and CV_A_) as follows: *I*_A_ = *V*_A_/mean^2^ = (CV_A_/100)^2^; CV_A_ = 100 × (sqrt(*V*_A_)/mean).

## 3. Results

Rates of F_1_ fanning changed over time (linear mixed-effects regression model; day: *β* = 22.59, s.e. = 7.58, *t*_47.39_ = 2.98, *p* = 0.0045; day^2^: *β* = −3.79, s.e. = 1.15, *t*_35.86_ = −3.31, *p* = 0.0022); fanning increased over time after fertilization and then decreased shortly after the fry hatched (figure 1). On average, rates of fanning were higher in sons than in fathers on ED5 (table 1), but the difference was not statistically significant (*t*_34.8_ = 1.36, *p* = 0.18).

**Table 1.** Means and variances of time fanning on the fifth day after fertilization (ED5).

	mean (s)	variance
fathers	28.77	1558.36
sons	51.12	3954.03
offspring family means	38.88	2542.15
animal model^a^	39.47	3280.78
animal model (corrected)^a^	41.55	3543.40

^a^The mean given here is the predicted value. The variance is the total variance from the posterior distribution.

**Figure 2.**
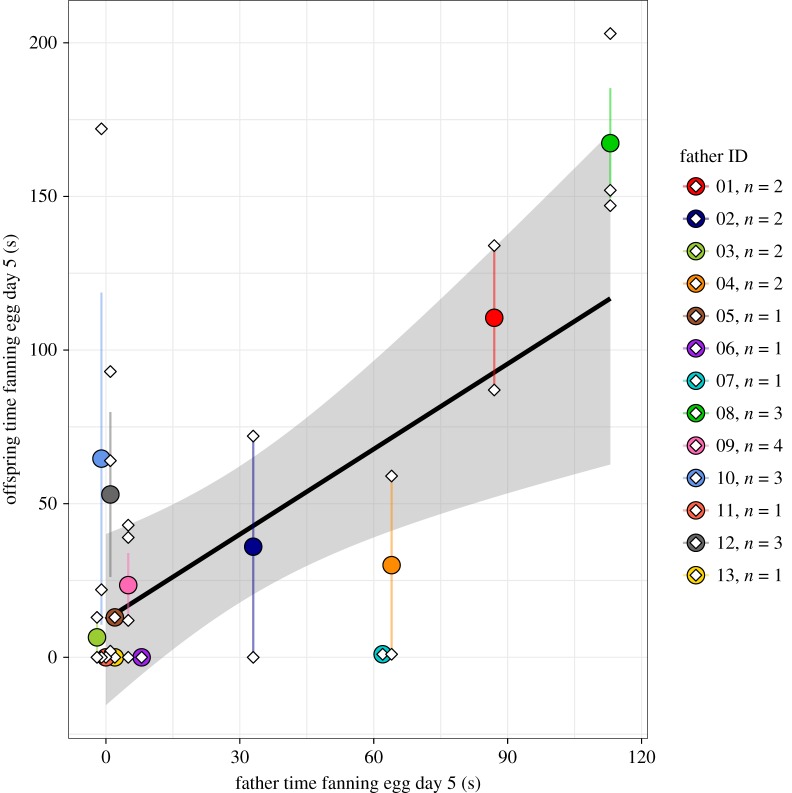
Relationship between fanning in fathers and sons. Son fanning time plotted against father fanning time. For visualization, the regression line is in black and 95% CI are indicated in grey (linear regression: *β* = 0.92 ± 0.27, *t*_11_ = 3.48, *p* = 0.005). Solid circles and lines indicate means ± s.e. for each family. Individual data points are indicated with diamonds. Legend shows the number of offspring per family, and colour coded as in figure 1.

We focused on the peak of fanning at ED5 for our heritability analyses. Means and variances of fanning on ED5 are in table 1. There was a close correspondence between the behaviour of fathers and their sons. Specifically, there was a strong relationship between father fanning on ED5 and the mean of their sons' fanning on ED5 (figure 2). Indeed, according to the animal model, the heritability of fanning on ED5 was *h*^2^ = 0.96, CI = 0.72–1.00. The heritability estimate remained high after controlling for the effect of male condition (weight/length), clutch size and ‘patch’ (common rearing environment): *h*^2^ = 0.90, CI = 0.36–1.00. Neither covariate was a significant predictor of fanning (condition: pMCMC = 0.55, clutch size: pMCMC = 0.62). Moreover, the variance accounted for by patch was very small (2.35% of variance explained with a CI of 1.33–3.49%).

To address the possibility that stochastic variance during the short 5 min observation might have influenced our heritability estimate, we estimated the repeatability of time fanning across two 5 min observation periods. The repeatability estimate was very high: 0.93 with a 95% credible interval of 0.51–0.99. This result strongly suggests that males maintain their rank order differences in fanning over the course of day 5, and that fanning does not change dramatically within males on day 5. Moreover, when we simulated deviations that might have occurred due to sampling error, the heritability estimates ranged from 0.17 to 0.98, and are strongly left-skewed (95% of the estimates are greater than 0.61), which suggests that our results are robust to stochastic variation from sampling error (electronic supplementary material, figure S1).

Heritabilities, evolvability and the coefficient of genetic variation are presented in table 2. Non-zero estimates of *I*_A_ and CV_A_ suggest that parenting behaviour can readily evolve in stickleback (table 2).

**Table 2.** Quantitative genetic estimates of time fanning on the fifth day after fertilization (ED5) from the animal model. The ‘corrected’ estimates control for the effects of male condition and clutch size.

estimation technique	*h*^2^	*V*_A_	*I*_A_	CV_A_
animal model (uncorrected)	0.96 (CI = 0.72–1.0)	3142.21	2.02^a^	142.02^a^
animal model (corrected)	0.90 (CI = 0.36–1.0)	3192.59	1.82^a^	134.90^a^

^a^Based on the predicted mean.

## 4. Discussion

Here, we show that our measure of parenting behaviour is highly heritable in three-spined stickleback. Despite variation within families and a relatively small sample size, our heritability estimates are significantly different from zero, and higher than reported estimates of heritability of parenting behaviour in other species [2–7]. Other studies have focused on the heritability of offspring provisioning behaviour, which involves dynamic interactions between parents and offspring. Fanning is a form of direct care that clearly changes over time (figure 1), but is unlikely to be influenced by the behaviour of the embryos, which might also help explain why our estimates were relatively high compared to estimates of heritability of provisioning behaviour. Variable levels of begging by offspring, for example, could influence rates of provisioning behaviour and therefore contribute additional environmental variance.

Heritabilities close to 1 suggest a very simple genetic basis, i.e. the trait is influenced by a few genes of large effect. However, there are at least three other non-exclusive explanations for the high heritability estimate. First, the small sample size might have increased the likelihood that an extremely high heritability estimate was obtained by chance alone [39]. While a larger sample size would have been preferable, unfortunately obtaining parenting data on a large scale in this system is challenging. Second, theory predicts that because natural selection removes genetic variation, traits under weak selection are expected to be more heritable than traits under strong selection; therefore, one explanation for the high heritability estimate is that variation in fanning is not under selection. This explanation seems unlikely given extensive population-level variation in fanning behaviour among stickleback populations [40], and that fish adjust fanning in response to changes in the environment [20–24]. That being said, it is possible that parenting behaviour in male sticklebacks is under weaker selection than parental behaviour in biparental species because it is only expressed in males [7].

Finally, it is possible that environmental effects contributed to the resemblance between sons and their fathers in this study. For example, F_1_ offspring received care from their fathers up to the day of hatching, leading to the possibility of non-genetic paternal effects. Previous studies have shown that variation in parenting can influence offspring traits in rodents [41–44] and in stickleback [30,45–48], potentially introducing environmental, non-genetic effects of parents on offspring that could increase the resemblance between parents and offspring. In rats and monkeys, offspring learn their parenting style from their parents during postnatal interactions with them [33,34], but there is no evidence to date that offspring learn parenting behaviour as embryos (when F_1_ in this study received parental care from their fathers). Another possible environmental cause of resemblance between fathers and sons is overall quality or condition: fathers might fan more because they are in good condition and therefore produce sons in good condition that also fan more. However, we did not detect an effect of condition, clutch size or rearing environment on fanning behaviour, which suggests that these environmental effects are not contributing to our results. Finally, environmental covariance between parents and offspring can inflate estimates of additive genetic variation [49], and this common environmental effect can be greater in magnitude than a genetic effect [50]. Importantly, conditions were controlled as much as possible in this laboratory study in order to minimize environmental variance.

This study provides some evidence for genetic variation in a dynamic, environmentally responsive behaviour (fanning). Indeed, levels of fanning in the laboratory-reared F_1_ population increased up until hatching and then declined over time. This pattern is consistent with observations of male stickleback fanning behaviour in the field and in the laboratory [16,17,21,23]. Despite changes in behaviour over time, individual differences in fanning behaviour at ED5 are heritable in this system.

The finding that parenting behaviour is heritable in male stickleback has several evolutionary implications. First, parenting behaviour is a paradigmatic example of indirect genetic effects (IGEs)—when traits in one individual are influenced by genes in another individual—and IGEs can influence predictions about how traits respond to selection [51]. Second, a highly heritable component to parenting behaviour could have implications for sexual selection. Male stickleback advertise their parental abilities during courtship, e.g. by fanning [52], and a previous study found that individual differences in fanning behaviour are maintained even during courtship [17]. Theory predicts that such male advertisement of care during courtship can be reliable when parental care is costly and necessary for offspring survival [53]. Finally, assuming that quantitative variation in parental care has fitness consequences, certain forces must be maintaining the genetic variation within natural populations. One possibility might be a fitness trade-off, where individuals that provide high levels of parental care, for example, are less aggressive and/or less effective at attracting mates. However, a previous study found that high levels of parenting behaviour do not come at the expense of territorial defence or mate attraction [17]. Other forces such as negative frequency-dependent selection, spatio-temporal variation in selection pressures or G × E interactions might play an important role in maintaining genetic variation in this trait.

In conclusion, our results suggest that parenting behaviour is heritable in male stickleback, which suggests that there is ample opportunity for parenting behaviour to evolve in this system. Indeed, it is intriguing to consider how paternal care behaviour might have facilitated the adaptive radiation of stickleback because fathers might be able to buffer their offspring from environmental variation, thereby allowing colonists to persist in a new environment [40]. We know surprisingly little about variation in parenting among populations of stickleback, but males in certain populations of stickleback build nests but do not provide care for their embryos and fry [54–59]. Our findings suggest that this variation is tractable for further genetic dissection.

## Supplementary Material

Supplementary figure 1

## Data accessibility

Data are available on Dryad (http://dx.doi.org/10.5061/dryad.rm230) [60].
