# Correction to ‘Parenting behaviour is highly heritable in male stickleback’

**DOI:** 10.1098/rsos.191372

**Published:** 2019-08-21

**Authors:** Alison M. Bell, Rebecca Trapp, Jason Keagy

*R. Soc. open sci.* **5** 171029. (Published Online 10 January 2018) (doi:10.1098/rsos.171029)

The Dryad link given in the data accessibility statement of this article is incorrect. The correct link is as follows: https://doi.org/10.5061/dryad.t2ch6.2.

