# Supplementary material for: Parenting behaviour is highly heritable in male stickleback
Source: R Soc Open Sci. 2018 Jan 10;5(1):171029. doi: 10.1098/rsos.171029 (PMC5792893; doi:10.1098/rsos.171029)
Supplement: Supplementary figure 1 [file rsos171029supp1.docx]

**Supplemental Figure 1. Simulation of stochastic variation from sampling error.** In a separate study, we recorded the behavior of one male for one hour on Day 5 and then sampled at 5-minute intervals, which allowed us to calculate for each 5-minute sample the deviation from the proportion of time fanning occurred across the entire hour. We used these data to simulate deviations that may occur due to sampling error and added these deviations to each data point, under the condition that fanning was not allowed to be less than 0 or greater than 300 seconds. We then recalculated the heritability estimate from the animal model. This was then repeated 1,000 times to give a range of heritability estimates expected if the deviations were as large as those observed in our 11 five-minute sessions. The distribution of heritability estimates is displayed as a histogram. The heritability estimate from the actual data presented in the paper is indicated by the red horizontal dashed line.
